# Primary Neuroendocrine Carcinoma of the Ileum With Markedly Elevated Carcinoembryonic Antigen (CEA) Levels: A Case Report

**DOI:** 10.7759/cureus.66676

**Published:** 2024-08-12

**Authors:** Aya Sugimoto, Tsutomu Nishida, Kana Hosokawa, Yoshifumi Fujii, Dai Nakamatsu, Kengo Matsumoto, Masashi Yamamoto, Koji Fukui

**Affiliations:** 1 Department of Gastroenterology, Toyonaka Municipal Hospital, Toyonaka, JPN

**Keywords:** radiotherapy (rt), somatostatin receptor scintigraphy, somatostatin receptor type 2, cervical lymph node metastasis, neuroendocrine carcinoma(nec), elevated carcinoembryonic antigen (cea), carcinoembryonic antigen (cea)

## Abstract

Neuroendocrine carcinomas (NECs) are rare and highly malignant tumors with a generally poor prognosis. Carcinoembryonic antigen (CEA) is often associated with adenocarcinoma, but its significant elevation in NEC cases is unusual. A 69-year-old man was admitted to our hospital in January 2016 due to syncope induced by anemia. The patient had a hemoglobin level of 8.0 g/dL and an ileocecal mass causing small bowel obstruction on computed tomography. His CEA level was markedly elevated at 3625.4 ng/mL. A colonoscopy revealed a neoplastic lesion in the terminal ileum, leading to an emergency ileocecal resection. Pathology confirmed a NEC, positive for synaptophysin and CEA, with a Ki-67 index of 30%. The patient was diagnosed with stage IIIb NEC (pT3N2M0). A postoperative increase in CEA to 4124.6 ng/mL and metastases in the right lung and multiple lymph nodes were detected. Initial chemotherapy with irinotecan, cisplatin (IP), and octreotide acetate proved ineffective. Subsequent octreoscans showed disease progression. Switching to everolimus as second-line therapy temporarily decreased CEA levels and tumor size, but the disease progressed with cervical lymph node involvement. The patient underwent palliative radiotherapy but succumbed to disease progression in May 2018, with a final CEA level of 36,643 ng/mL. Necropsy of the cervical lymph nodes was consistent with the original surgical findings. This case highlights the aggressive nature and challenging management of NEC with significantly elevated CEA levels.

## Introduction

Carcinoembryonic antigen (CEA) is a tumor marker commonly associated with adenocarcinoma. This glycoprotein is expressed primarily in fetal digestive organs and is normally found at low levels in adults. However, in these cancers, CEA is abnormally expressed and released into the bloodstream [1].

Neuroendocrine carcinomas (NECs) represent 10-20% of all neuroendocrine neoplasms (NENs) and are characterized by poor differentiation and an overall unfavorable prognosis. According to the European Society for Medical Oncology (ESMO) guidelines [2], NECs are distinct entities from well-differentiated neuroendocrine tumors (NETs) G3, as defined by the World Health Organization (WHO) classifications in 2017 and 2019. This distinction is based on the high proliferative activity of NECs (Ki-67 > 20%) and the marked differences in prognosis between these subtypes. Clinically, NECs pose significant challenges: surgical interventions are often limited to primary tumor resection or palliative debulking, while systemic chemotherapy, although recommended, yields only short median overall survival (11-19 months) despite relatively high response rates (30-67%). Furthermore, the absence of established second-line therapies and the potential for late recurrences, even after 10-20 years, necessitates a comprehensive and long-term management approach.

According to the 2019 WHO classification, tumors that contain both neuroendocrine and non-neuroendocrine components, each greater than 30%, are called mixed neuroendocrine-non-neuroendocrine neoplasms (MiNENs) [3]. In these tumors, the degree of malignancy of each component is not specified. When epithelial tumors contain an adenocarcinoma component, there is no specific tumor marker, although elevated CEA values may be observed [4]. 

In the present case, a marked increase in CEA levels was associated with a diagnosis of ileal NEC in the resected specimen. No adenocarcinoma component was identified in this case, and CEA production by the tumor cells was confirmed by immunostaining. An increase in CEA levels was also observed at the time of recurrence, but no adenocarcinoma component was found in the metastatic recurrence. We report this unique case in detail.

## Case presentation

**Figure 1:**
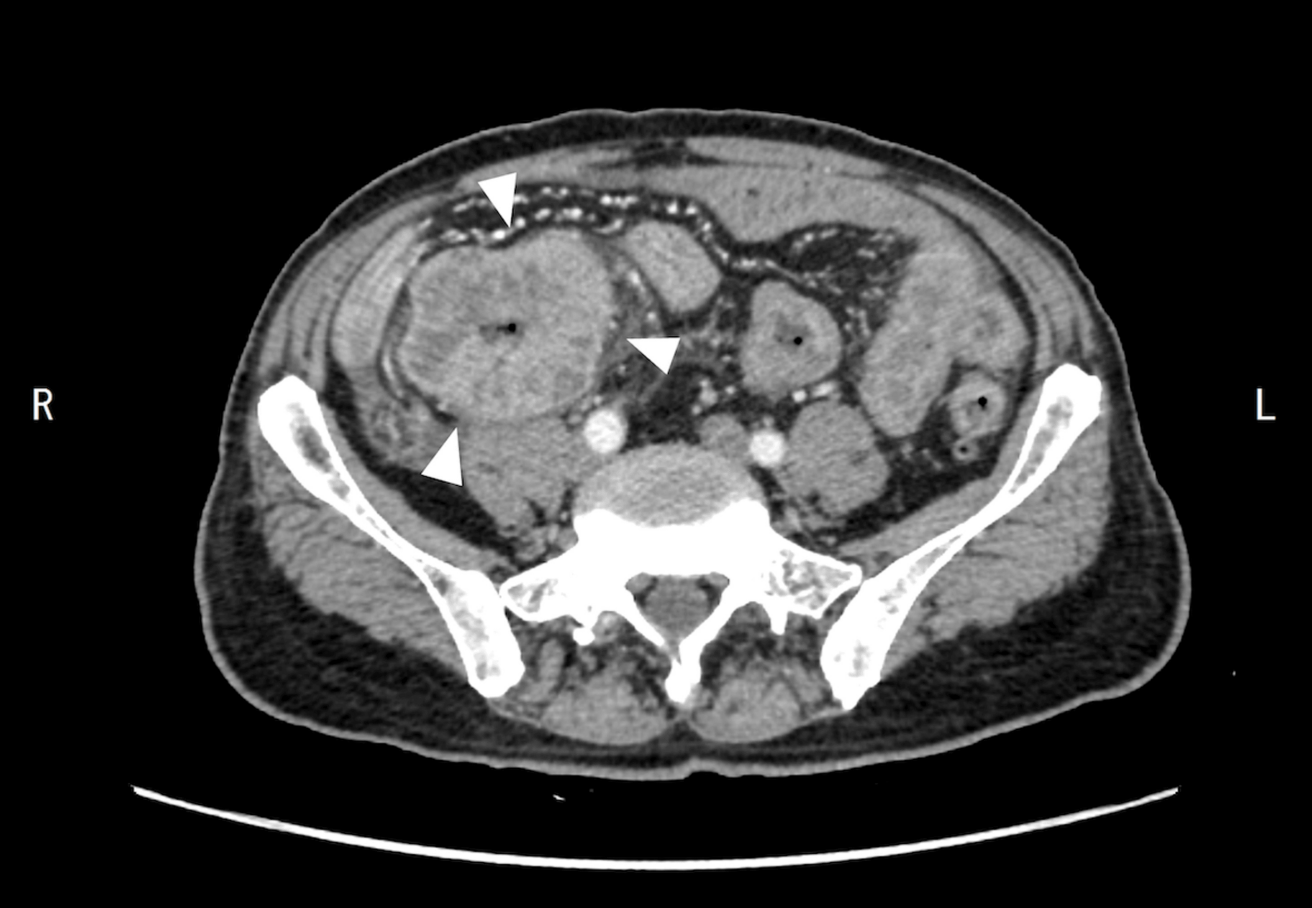
Abdominal computed tomography Abdominal computed tomography shows an ileocecal mass causing small bowel obstruction. The white triangles indicate the location of the tumor.

In January 2016, a 69-year-old man presented to his primary care physician with syncope due to anemia. The patient had no significant past medical history or comorbidities that could have influenced his condition. An abdominal computed tomography (CT) scan revealed a small bowel obstruction caused by an ileocecal mass (Figure 1).

The patient was referred to our hospital for further evaluation. Laboratory tests revealed anemia (hemoglobin level of 8.0 g/dL) and a markedly elevated carcinoembryonic antigen (CEA) of 3,625.4 ng/mL (Table 1).

**Table 1:** Laboratory data on admission

Parameters	On admission	Reference range
White cell count (/μL)	6000	3300-8600
Neutrophils (%)	59	40-68
Lymphocytes (%)	33.5	26.0-46.6
Red blood cells (×10^4^/μL)	358	389-492
Hemoglobin (g/dL)	8.0	11.6-14.8
Hematocrit (%)	25.9	35.1-44.4
Platelet count (×10^4^/μL)	44.7	15.8-34.8
Prothrombin time (PT) (%)	72	70-130
PT-International normalized ratio	1.15	0.9-1.1
Total protein (g/dL)	6.6	6.6-8.1
Albumin (g/dL)	2.5	4.1-5.1
Aspartate transaminase (U/L)	17	13-30
Alanine transaminase (U/L)	10	7-23
Lactate dehydrogenase (U/L)	212	135-214
Alkaline phosphatase (U/L)	217	35-104
Amylase (U/L)	43	44-132
Blood urea nitrogen (mg/dL)	15	8-20
Creatinine (mg/dL)	0.92	0.46-0.79
Fasting Glucose (mg/dL)	94	73-109
Sodium (mEq/L)	138	138-145
Calcium (mg/dL)	8.2	8.8-10.1
Total bilirubin (mg/dL)	0.67	0.2-1.2
C-reactive protein (mg/dL)	3.35	< 0.3
Carcinoembryonic antigen (ng/mL)	3625.4	< 5
Carbohydrate antigen 19-9 (U/mL)	14	< 37
Soluble interleukin-2 receptor (U/mL)	698	156.6-474

**Figure 2:**
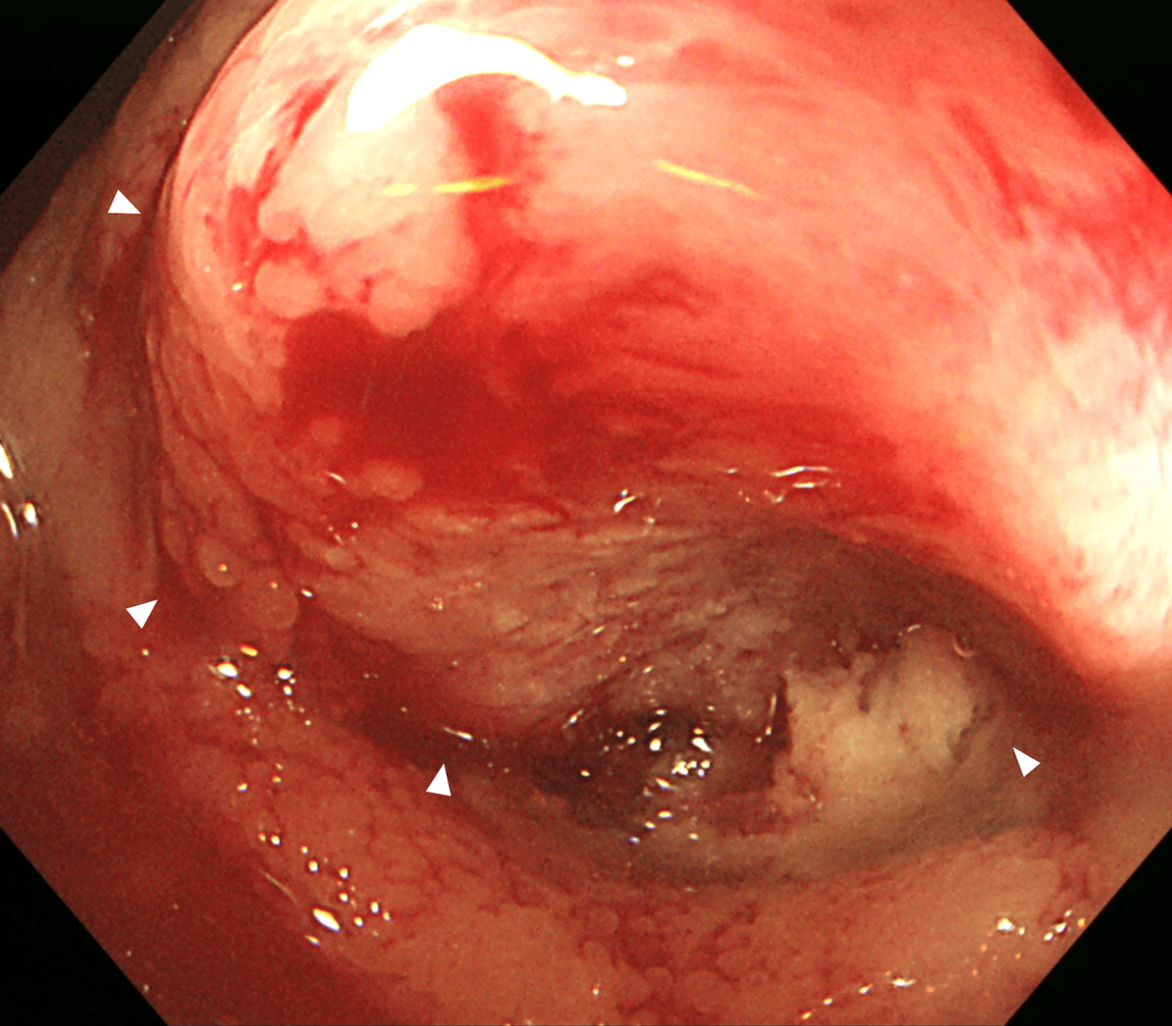
Colonoscopy image Colonoscopy image showing a circumferential neoplastic lesion in the terminal ileum. The white triangles indicate the tumor.

A colonoscopy revealed a circumferential neoplastic lesion in the terminal ileum that was difficult to pass through the scope (Figure 2).

**Figure 3:**
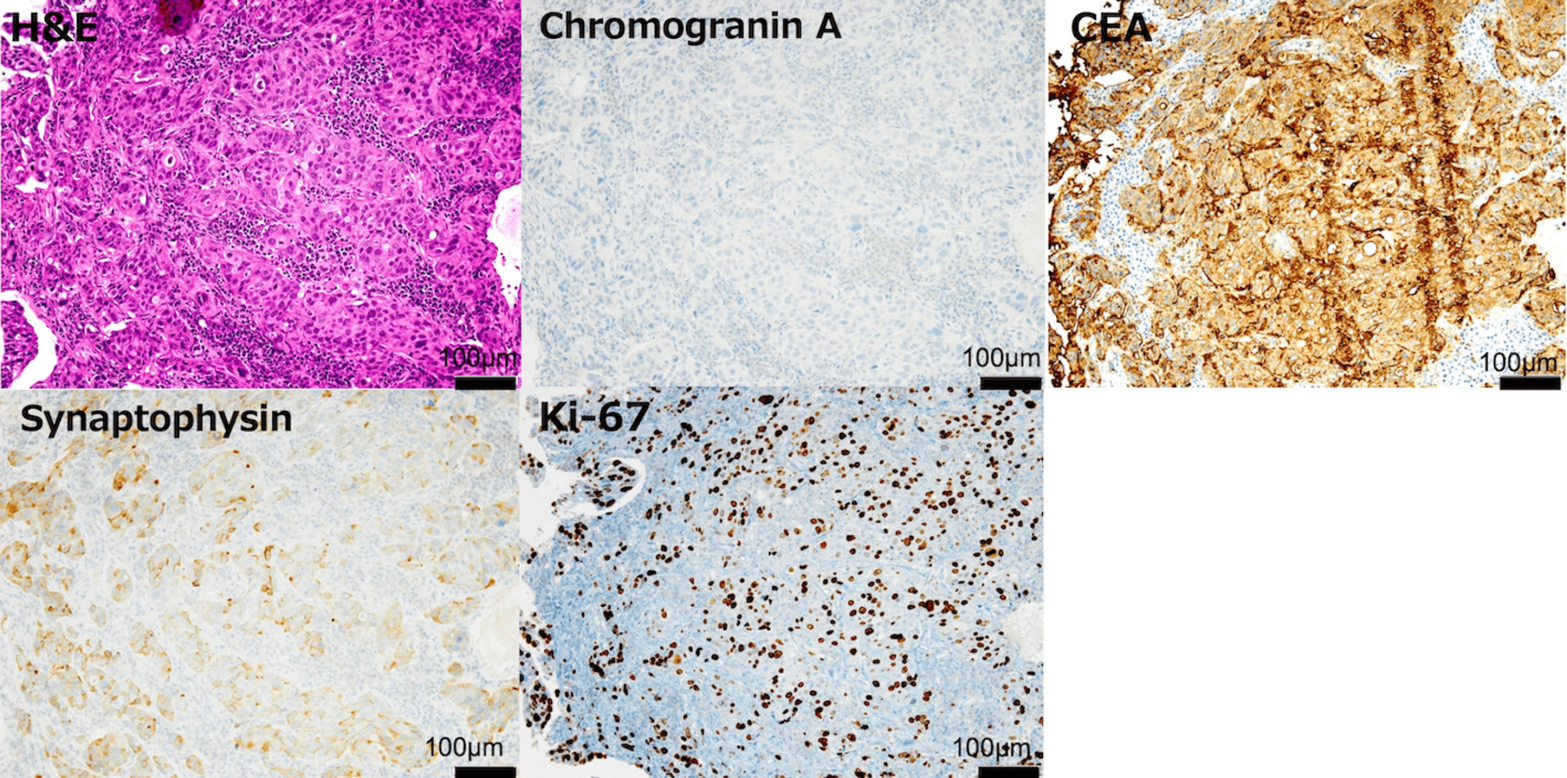
Pathological findings Pathological findings illustrating neuroendocrine carcinoma (NEC) with immunohistochemical results for synaptophysin, CEA, and Ki-67.

He was diagnosed with ileal obstruction due to an ileal tumor and underwent emergency ileocecal resection. Pathology revealed a neuroendocrine carcinoma (NEC) that was positive for synaptophysin and CEA, with a Ki-67 index of 30% (Figure 3), and negative for chromogranin A and CD56.

Interestingly, while the NEC component was present, there was a complete absence of adenocarcinoma in the resected specimen. The patient was ultimately diagnosed with pT3N2M0 stage IIIb NEC. The patient was followed without neoadjuvant chemotherapy due to insufficient evidence at that time.

**Figure 4:**
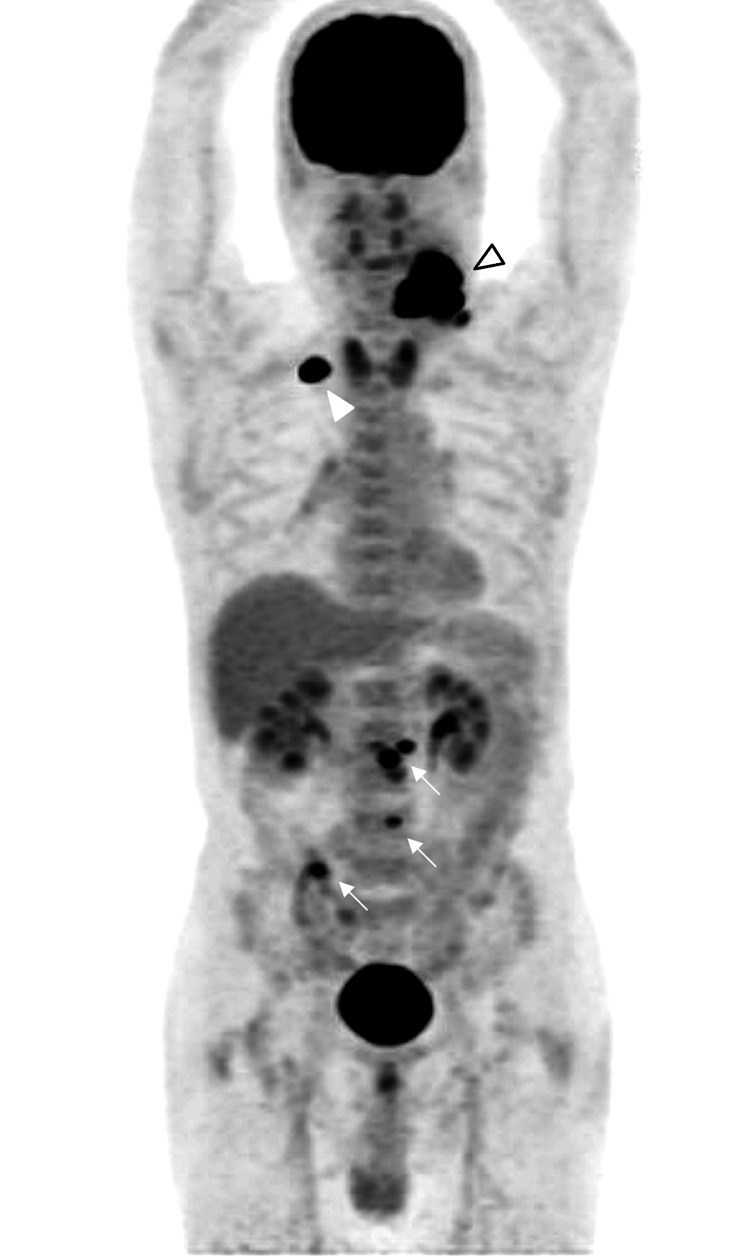
Positron emission tomography (PET) with FDG integration A coronal view of a positron emission tomography (PET) scan with FDG integration shows a right lung metastasis (white triangle), cervical lymph node metastases (black framed white triangle), and multiple lymph node metastases (arrows).

CEA levels increased to 4,124.6 ng/mL 5 months after surgery. The patient was suspected to have a recurrence. Positron emission tomography with 2-deoxy-2-(fluorine-18) fluorodeoxyglucose (FDG) integrated with CT showed metastases in the right lung and multiple metastases in the left subclavian, left cervical, and paratracheal lymph nodes (Figure 4).

**Figure 5:**
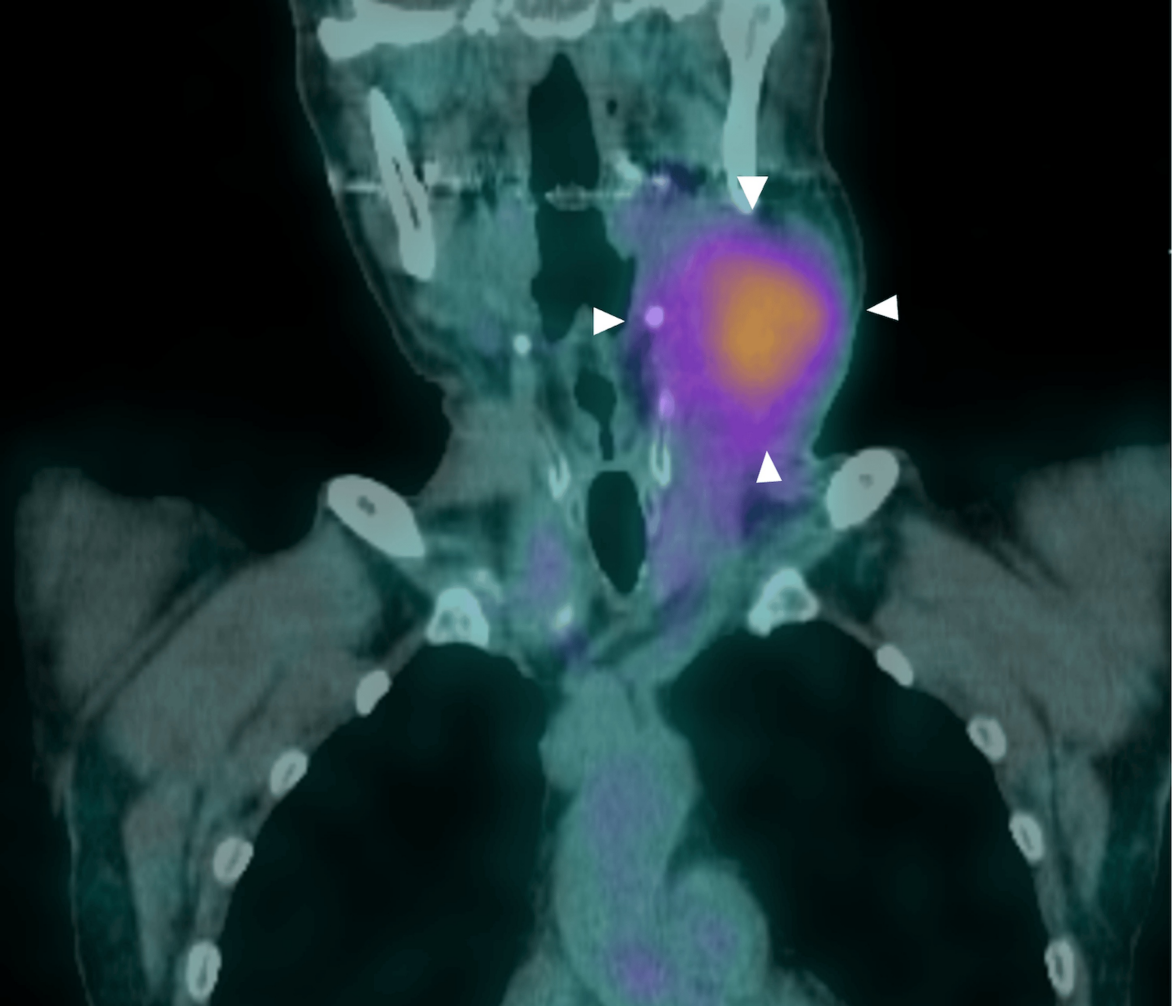
Somatostatin receptor scintigraphy Somatostatin receptor scintigraphy showing uptake in cervical lymph node metastasis (white triangles).

In August 2016, the CEA level increased to 13,898.1 ng/mL. The patient had a good performance status of 0 and no significant comorbidities. Additional immunohistochemistry studies from other institutions showed both somatostatin receptor type 2 (SSTR2) and SSTR5 with a score of 0, but positive for mTOR (imaging not available). Somatostatin receptor scintigraphy (SRS) showed uptake in the right lung metastases and multiple lymph nodes with a Krenning score of 3 (Figure 5).

**Figure 6:**
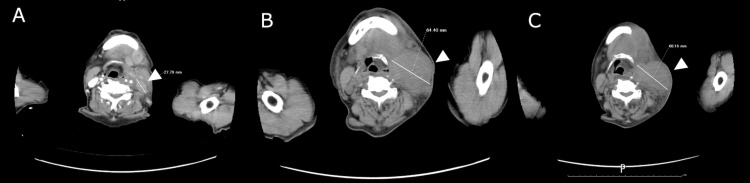
Serial computed tomography scans of cervical lymph node metastases Serial computed tomography scans of cervical lymph node metastases (white triangles): (A) at recurrence diagnosis in May 2016, (B) before radiotherapy in August 2017, (C) at the time of adopting best supportive care in February 2018.

**Figure 7:**
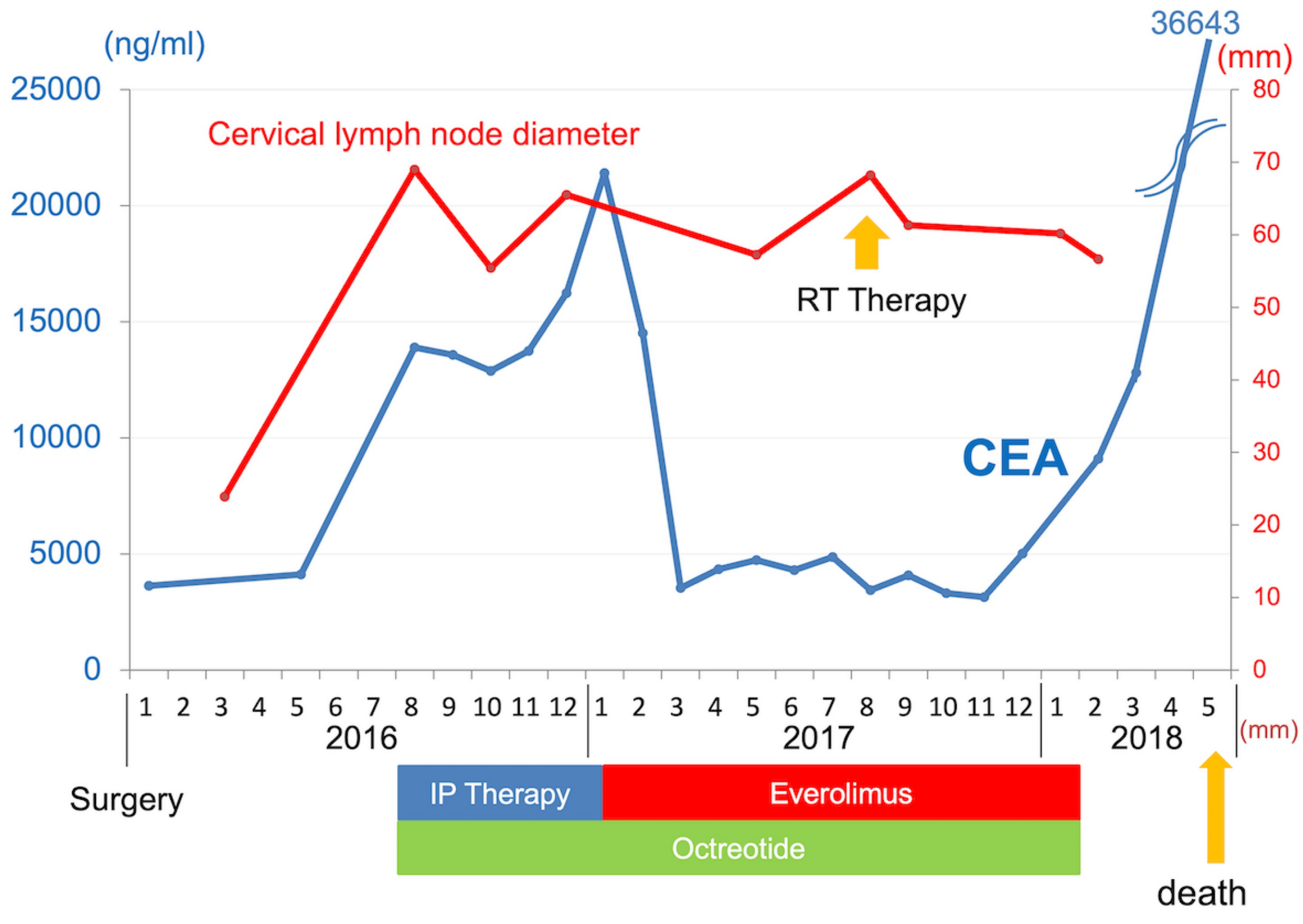
Clinical course Clinical course, summarizing the patient's treatment journey, and disease progression.

Therefore, we administered long-acting release (LAR) octreotide acetate along with chemotherapy. Chemotherapy was started with irinotecan plus cisplatin (IP). At the end of the third course of IP therapy, the cervical lymph node metastases shrank, and the associated pain improved. Based on the CT evaluation, we judged the efficacy of chemotherapy as a stable disease with a 21% tumor reduction, as measured according to RECIST [5]. However, after six courses of IP therapy, CT showed disease progression, and the CEA level increased to 21,410.7 ng/mL. We judged disease progression against IP with octreotide acetate. Renal dysfunction occurred with a creatinine level of 1.48 mg/dL and eGFR of 37.3 mL/min/1.73 m² due to cisplatin toxicity. As second-line therapy, due to positive mTOR expression, everolimus in combination with octreotide acetate LAR was started in January 2017, with significant tumor shrinkage, and CEA level decreased to 3,147.2 ng/mL. However, in August 2017, the cervical lymph nodes increased (Figure 6), and painfully compressed the trachea. The patient underwent an emergency tracheostomy and was treated with 30 Gy/10 Fr radiotherapy for pain relief, which resulted in tumor reduction. He then received the best supportive care and died of tumor progression in May 2018 (Figure 7).

**Figure 8:**
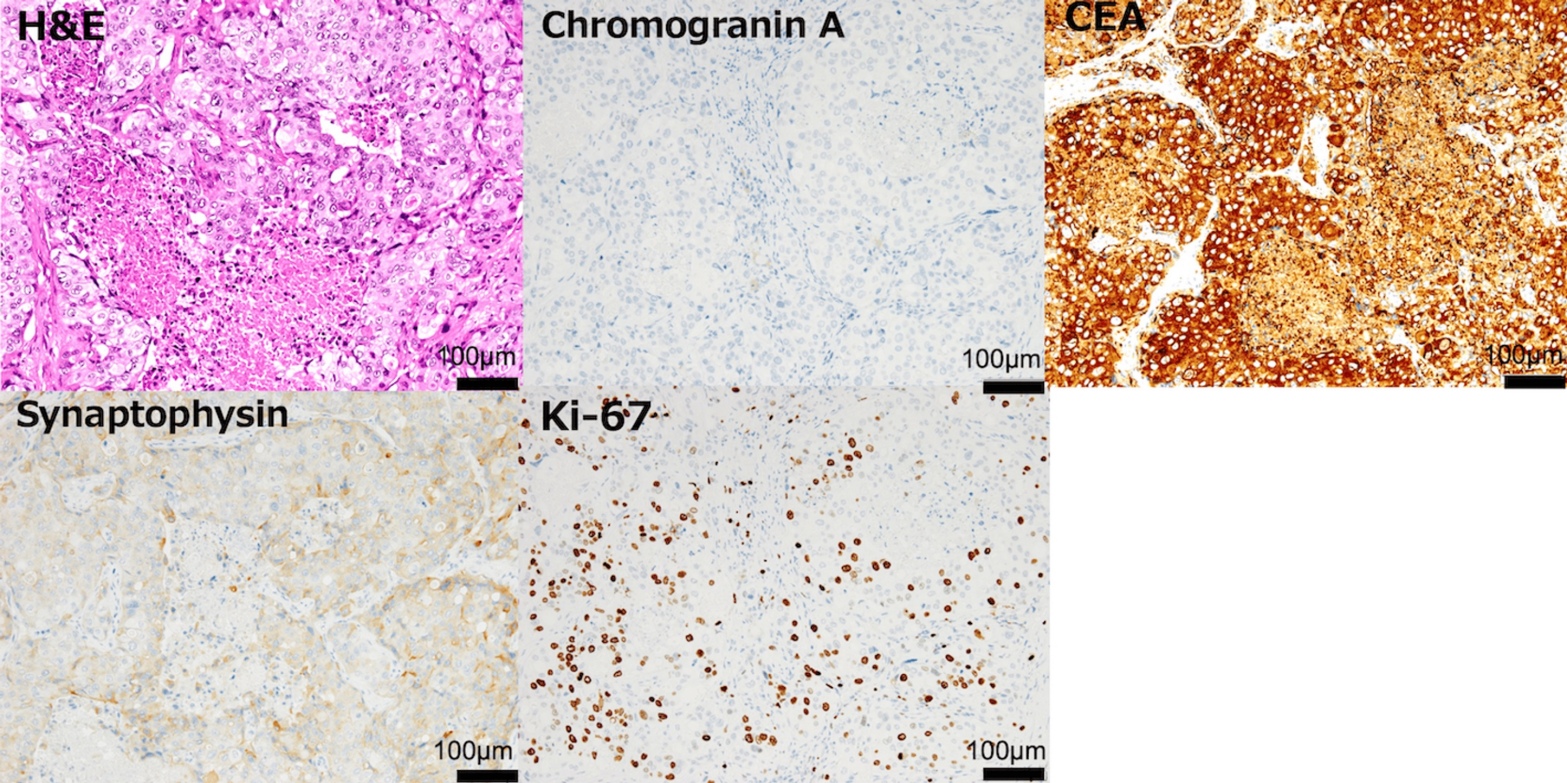
Necropsy of the cervical lymph nodes The necropsy of the cervical lymph nodes resembled the pathological features of the surgical specimen.

Eventually, the CEA level increased to 36,643 ng/mL. The necropsy of the cervical lymph node was similar to that of the surgical specimen (Figure 8).

## Discussion

Gastroenteropancreatic NECs, the poorly differentiated types, exhibit aggressive behaviors. The 2010 WHO classification system categorized neuroendocrine tumors (NETs) based on their proliferative capacity, regardless of histological appearance, as NET G1 and G2 for lower proliferative rates and NEC G3 for higher rates (Ki-67 index > 20%). Subsequent updated WHO classification identified well-differentiated NETs with Ki-67 index > 20%, leading to their classification as both NET G3 and NEC. The terminology of the tumor itself was also unified from NET to neuroendocrine neoplasm (NEN). However, the term NET continues to be used in the pathological classification. These tumors are genetically distinct; NET G3 often presents ATRX/DAXX mutations and lacks the p53 and Rb mutations commonly found in NEC [6]. The guidelines suggest the use of MEN1/ATRX/DAXX and RB1/TP53 to differentiate NET G3 from NEC [2,7]. As a result, NEC tends to spread early and widely. Localized cases can sometimes be cured with intensive treatment, but the recurrence rate is high, and the overall prognosis remains poor. Treatment plans should be tailored to the individual and developed in consultation with a multidisciplinary team [8]. A report by Gao et al. [9] suggests that serum CEA levels are significantly higher in NECs compared to NETs G3. This finding suggests that CEA can be used as a biomarker to distinguish NECs from NETs G3. They also reported that elevated levels of CEA are associated with poorer survival outcomes in patients with NENs [9].

Small bowel NENs are the most common cancers of the small bowel, with a reported annual incidence of approximately 1 in 100,000 people. One study reported an annual incidence of 5.33 per 100,000 people and a prevalence of 581 per 100,000 based on the autopsy registry [10]. These tumors are often clinically undetected during life, and many are only discovered during autopsy. This finding suggests that the true prevalence may be higher. There are significant racial differences in NENs, with the highest incidence in black populations and the lowest incidence in Asian/Pacific Islanders. In Japan, small bowel NENs account for only 4% of all NENs.

NETs and NECs among the small bowel NENs show distinct differences in their biological behavior, histologic characteristics, and epidemiology. In our case, the patient with primary NEC of the ileum had extremely high CEA levels, which presents a unique diagnostic challenge. Although CEA is a well-known marker for adenocarcinoma, it is not commonly elevated in NENs, including NECs [9]. However, this case, with a peak CEA level of 36,643 ng/mL before death, is extremely rare. CEA-producing NECs have been well described in immunohistochemical studies [11,12]. They grow rapidly, metastasize easily, and have a poor prognosis. Although elevated CEA levels in NECs are rare, they are known to be associated with medullary carcinoma, a type of NEN. In particular, medullary thyroid carcinoma (MTC) has high CEA levels, and CEA measurement is key to the diagnosis of MTC [13]. According to the guidelines of the North American Society for Neuroendocrine Tumors, preoperative levels > 30 ng/mL indicate that the disease has spread beyond the thyroid gland [14]. 

High levels of CEA have been reported in various NECs, including breast (54.4 ng/mL) [15], gallbladder (723 ng/mL) [16], prostate (11.15 ng/mL) [17], and anal canal poorly differentiated adenocarcinoma with neuroendocrine features (809.4 ng/mL)[18], and glucagonoma in the pancreas (660 ng/mL) [19]. This phenomenon is rare but important for diagnosis and monitoring. Egashira et al. found elevated CEA levels in five of 14 (35.7%) esophageal NEC cases, ranging from 5.3 to 19.6 ng/mL [20]. Thus, CEA elevation in NECs may be underrecognized. However, the peak CEA level in this case, 36,643 ng/mL just before death, is extremely unusual. The reason for this unusual expression is unclear but could involve changes in the tumor environment, genetic mutations, or unique differentiation within the tumor.

This case's aggressive nature and the complexity of its management, despite the treatments, underscore the need for more effective treatments for NEC, particularly for cases with unusual markers such as this one. The disease recurrence and progression in our patients, despite treatment, underscore the challenges in managing such cases and the importance of exploring new therapeutic options.

## Conclusions

In conclusion, this case highlights the rare but significant occurrence of markedly elevated serum CEA levels in NECs. Although it is known that NECs can produce CEA, reports of significantly high serum CEA levels are extremely rare. The exact mechanism behind this phenomenon remains unclear, but it suggests that NECs may shed CEA into the bloodstream, which may correlate with the malignancy and aggressiveness of the disease. This case highlights the need for clinicians to consider CEA as a potential indicator of disease severity in NECs. Further research is needed to understand the mechanisms driving CEA release in NECs and to develop improved diagnostic and therapeutic strategies for managing this aggressive cancer.
